# Nano-Antennas Based on Silicon-Gold Nanostructures

**DOI:** 10.1038/s41598-018-36851-w

**Published:** 2019-01-23

**Authors:** A. Kucherik, S. Kutrovskaya, A. Osipov, M. Gerke, I. Chestnov, S. Arakelian, A. S. Shalin, A. B. Evlyukhin, A. V. Kavokin

**Affiliations:** 1 Department of Physics and applied mathematics, Stoletov Vladimir State University, 600000 Gor’kii street 87, Vladimir, Russia; 2 Westlake University, 18 Shilongshan Road, 310000, Cloud Town, Xihu District, Hangzhou, China; 3 ITMO University, 49 Kronversky Ave., 197101 St. Petersburg, Russia; 4 Moscow Institute of Physics and Technology, 9 Institutsky Lane, Dolgoprudny, 141700 Russia

## Abstract

We experimentally realize nano-antennas based on hybrid silicon-gold nanoparticles (NPs). The silicon particles covered by clusters of small metal NPs are fabricated from a liquid phase under the effect of the laser irradiation. The complex nanoclusters containing both Si and Au components provide the enhancement of the near-field intensity and the resonant light scattering associated with excitation of multipole resonances in NPs. A strong sensitivity of the resonant light absorption to the hybrid particle size and material parameters is experimentally documented and theoretically discussed. The results demonstrate a high potentiality of the hybrid NPs for the realization of functional optical devices and metasurfaces.

## Introduction

Light scattering by micro- and nano-particles is at the origin of a rich variety of non-trivial optical effects that pave the way to multiple photonic applications. In this context, one of the most promising applications of NPs is for fabrication of nano-antennas able to transfer the electromagnetic field energy from near-field to far-field and vice versa on spatial scale of a few nanometers. One of the important challenges on the way to the optimization of such nano-antennas is the reduction of Ohmic losses. The Ohmic losses tend to be very high at the optical frequencies in metallic structures usually employed in nano-antennas^1^. Optical absorption and high Ohmic losses result in a significant heating that negatively affects the performance of these nano-antennas. On the other hand, certain dielectric and semiconductor nanostructures are characterized by much lower losses in the visible range that makes them suitable candidates for nano-antenna applications. In particular, it has been theoretically predicted^2^ and experimentally demonstrated that silicon NPs of a diameter of 100 and 200 nm possess well-resolved dipole resonances in the visible range^3,4^. The interplay between electric and magnetic multipole resonances in silicon NPs leads to a number of new effects such as the self-focusing of radiation, suppression of the back scattering etc^5^.

Combining the characteristics of noble metal and silicon NPs, one can potentially take advantage of the specific optical properties of both of them. In this context, hybrid silicon-metal NPs are especially promising as they allow for tailoring optical properties, plasmon resonances, electric and magnetic response functions.

Our work is aimed at the experimental demonstration of such tailoring and the enhancement of the near field emission of silicon NPs by covering them by small-size gold NPs. Golden shells trigger a remarkable nano-antenna effect that strongly affects the near field emission of silicon. We have already presented the suitable method on the formation of hybrid gold-silicon NPs with an on-demand size by laser irradiation of colloidal solutions^6^. The increase of the magnitude of NPs near field intensity is theoretically described in terms of multipole interaction in hybrid clusters triggered by the presence of golden nanoparticles. The remarkable resonant scattering properties of NPs are a consequence of the multipole effect as well.

## Results

### The Preparation of Colloidal Systems Baced on Nps

We have used the CW-laser ablation for creation of liquid colloidal systems containing Si or gold NPs and the additional pulse laser action to induce the hybrid cluster formation as described in refs^7,8^. The average size of synthesized NPs and its dispersion were tuned by variation of the parameters of laser irradiation^8^ and probed by the dynamic light scattering. Hereafter we discuss the possibility of using of spherical silicon NPs with diameters of 100 nm and 200 nm as cores of hybrid clusters, in particular. This is because these NPs are expected to show spectrally separated dipole resonances in the visible range. We argue that an uneven gold covering including Au particles of 10 nm should result in the increase of dipole-dipole interactions in the nanocluster, in general.

**Figure 1:**
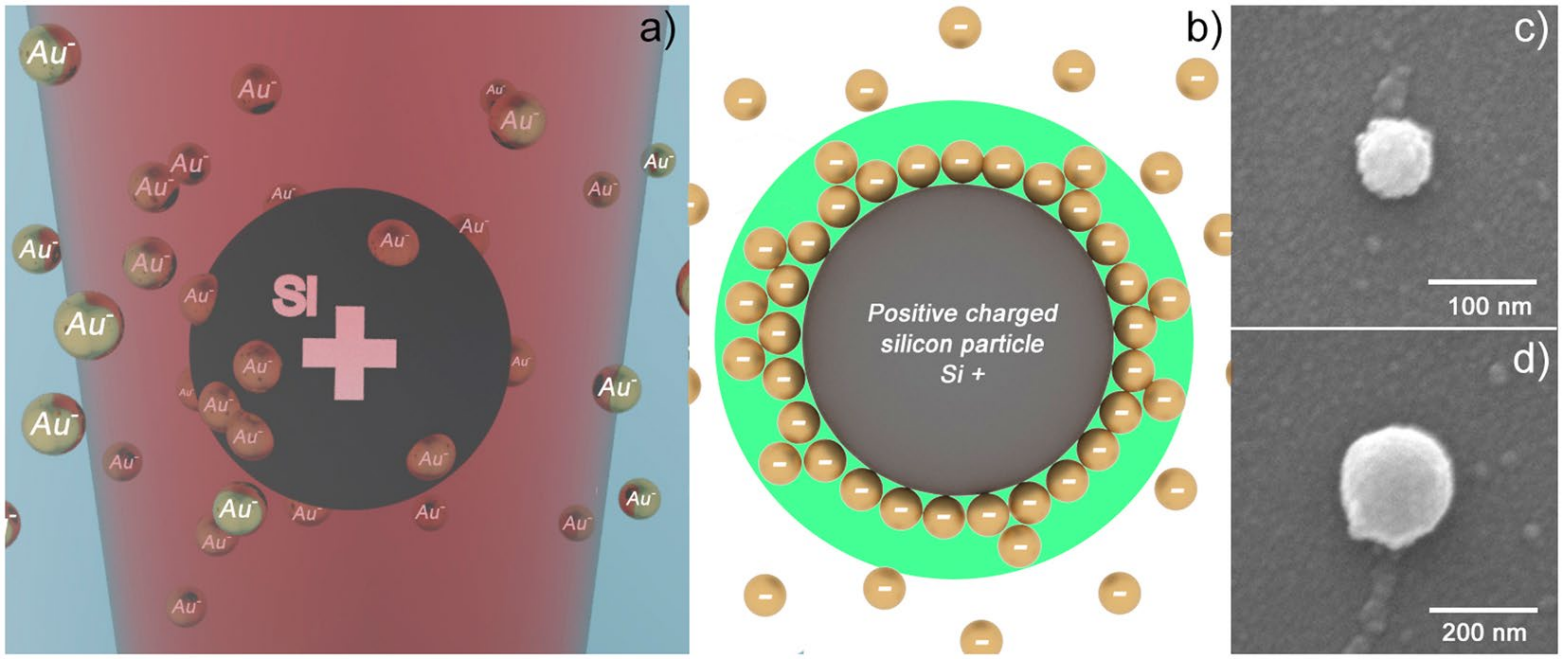
From left to right: the schematic image of the hybrid NPs formation under effect of the laser action on a liquid system consisting of Si and Au NPs (**a**); the scheme of the electrical double layer formation near the Si NP (**b**); the SEM-images of hybrid clusters composed by central silicon parts having a diameter of 100 nm and 200 nm and 10 nm gold nanoparticles covering the central silicon NP (**c**,**d**).

The physical mechanism of clusters’ formation is as follows (see Fig. 1). The gold NPs produced by laser ablation in liquid using the method described in ref.^8^ are characterized by a shortage of electrons. This shortage is revealed by the z-potential measurement. On the other hand, a colloidal system containing negatively charged NPs exhibits an electrolyte behavior^9^. The additional electrical charge is accumulated on the surfaces of the silicon NPs under the laser illumination. If there is a sufficient concentration of Au particles, their attraction to silicon NPs overcomes the electrostatic repulsion between equally charged NPs so that Si and Au particles start to agglomerate. After electron relaxation owing to the high surface charge density^10^, in the peripheral parts of the colloid the electrostatic repulsion is still dominant, so that the NPs are stabilized, and no further cluster growth takes place.

### Optical Study of Hybrid Nanoclusters

**Figure 2:**
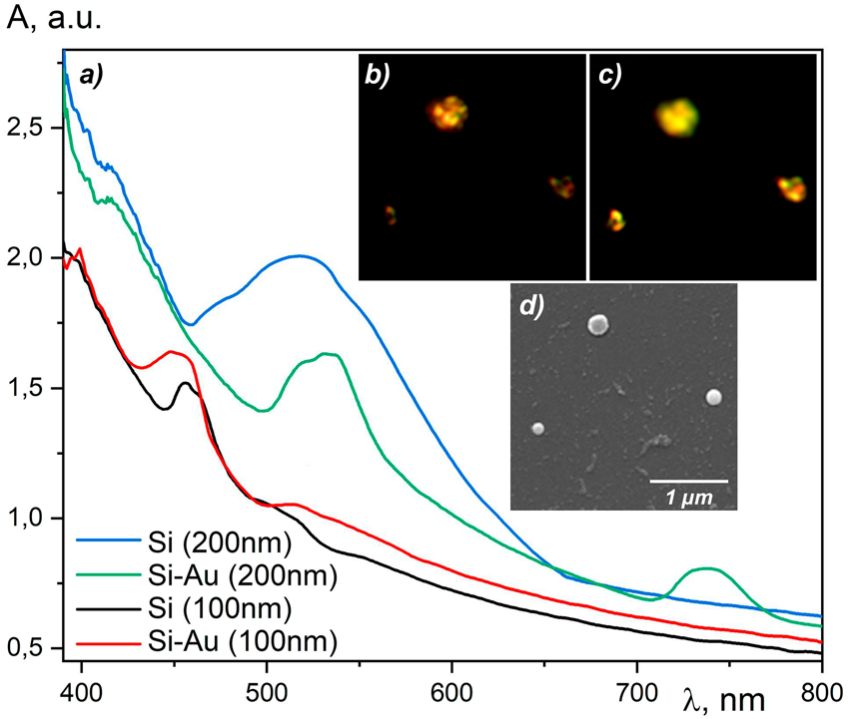
The absorption spectra (**a**) of liquid systems based on Si NPs of 100 nm (black curve) or 200 nm (blue curve) sizes, hybrid Au-Si nanoclusters with the silicon core of a diameter of 100 nm (red) and 200 nm (green curves); the inset subtracting a combined images (only the scattered light is collected) of the deposited hybrid gold-silicon nanoclusters for different illumination intensities: the white light intensity increases form 1000 lm (**b**) to 2000 lm (**c**). The SEM images of hybrid Si-Au NPs (**d**).

The initial Si colloidal systems demonstrate a resonant absorption in the spectral range of 450–500 nm for 100 nm NPs and 450–550 nm for 200 nm NPs (see Fig. 2). We detected wide absorption peaks caused by the NP size dispersion in the colloidal solution. Gold nanoparticles demonstrate plasmon resonances with the central wavelength of 523 nm.

After laser irradiation we observed dramatic changes of the optical properties of the colloidal systems. The spectroscopy investigation of the resulted liquid systems allows to identify the difference between absorption spectra of pure Si NPs and Si-Au cluster systems of the same sizes. This modification is caused by the cluster formation of silicon and gold NPs. The hybrid gold-silicon NPs are characterized by narrow amplitude peaks at about λ ~ 530 ± 30 nm and at about λ ~ 740 ± 30 nm (for Si-Au (200 nm) in Fig. 2) and by broad peak at about λ ~ 440 ± 20 nm and a broad shoulder at about λ ~ 500–600 nm (for Si-Au (100 nm) in Fig. 2). In the single particle approximation the absorption increases for Si-Au case as compared to the reference case of a pure silicon nanoparticle. The theoretical modeling results that are presented below fully reproduces these peculiarities. However, in the case of light scattering on a set of particles in a colloidal system, some more complex optical effects can be observed. They manifest themselves, in particular, in the strong variation of the absorption coefficient near the frequency of the plasmon resonance. It is important to note that NPs with a diameter of 100 and 200 nm demonstrate the increased absorbance in different spectral ranges. Clearly, the tailoring of hybrid silicon-gold nano-antennas offer a tool for tuning of the dipole resonance frequency in a wide spectral range.

We have deposited NPs using the technology of sputtering small colloidal drops on the solid substrate employing the method described in ref.^11^. The scanning electron microscopy (SEM) images of several hybrid nanoparticles are show in Fig. 2, the inserted panel (c). The peculiarity of deposited hybrid nanoparticles was emphasized by the dark-field technique^12^. The Mie-scattering in the wide spatial angle on the complex structures due to the illumination of the Si-Au clusters by the unfocused angled beam of a white light has been realized. We observe the changes of the central frequency of resonances from red to yellow spectral ranges (Fig. 2b,c). For the sake of comparison, we deposited clusters consisting of 100 nm and 200 nm Si-Au particles. Interestingly, using different levels of illuminating intensity one can observe various structural features of the nanoparticle clusters (Fig. 2b,c). At the low intensity of illumination one can see the light scattered by inhomogeneities of metal shells. However, this scattered signal vanishes with the increase of the illumination intensity (Fig. 2).

**Figure 3:**
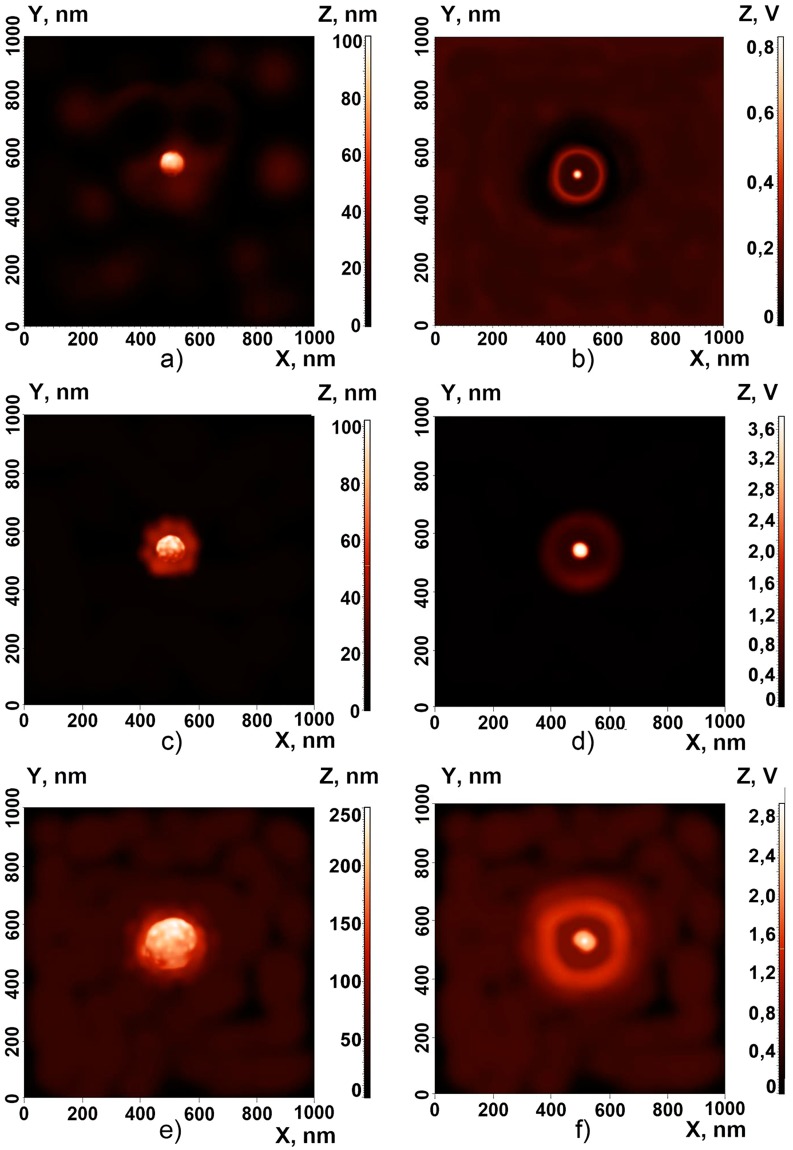
The images made by using atomic-force (**a,c,e**) and scanning near-field optical (**b,d**,**f**) microscopies of the pure Si particle of 100 nm diameter (top panels); 100 nm (medium panels) and 200 nm (bottom panels) Si-Au clusters.

Figure 3 allows one to compare the morphological sizes of clusters revealed by the atomic force microscope (AFM, the left panel) and the distribution of light intensities measured by the scanning near-field optical microscope (SNOM, the right panel). In the case of hybrid clusters the SNOM images are characterized by stronger peaks at the centers of the complex systems surrounded by weaker intensity crowns (see the SNOM profiles at the panels Fig. 3d,f). The increase of the near-field intensity in the hybrid cluster (Fig. 3d) compared to the pure Si NP (Fig. 3b) of the same size is achieved due to the plasmonic enhancement of light in the small metal nanoparticles located at the Si-core surface. It is important to note that the near-field images in Fig. 3 have been observed in the case of illumination at the wavelength of 514 nm that is close to the plasmon resonance of single gold NPs.

### Theoretical modelling

**Figure 4:**
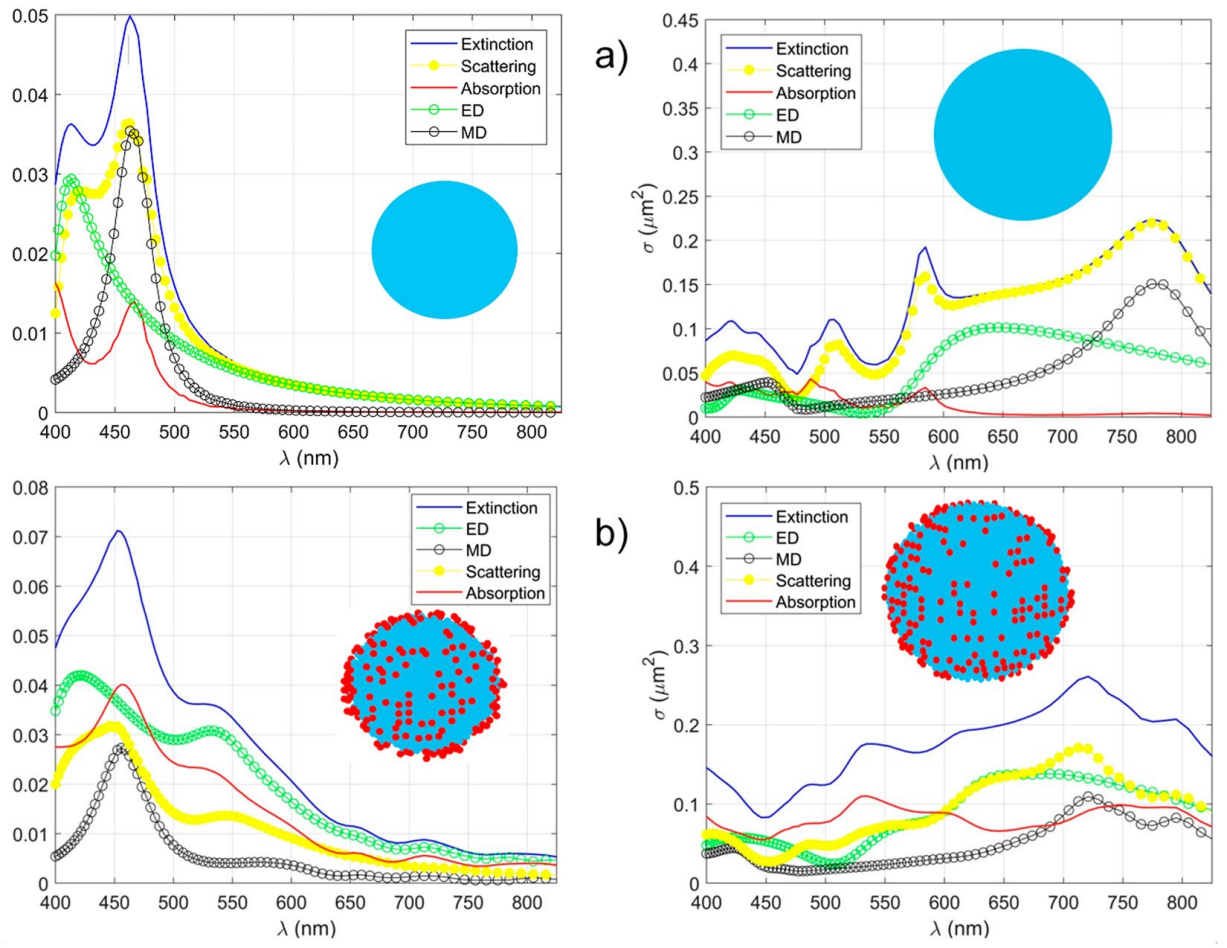
The spectra of the extinction, scattering and absorption cross sections calculated for (**a**) pure spherical Si-particles, (**b**) the Si-particles covered by random clusters of Au-particles. The left hand panels correspond to the 100 nm Si-particles, the right hand panels correspond to 200 nm Si-nanoparticles. The diameter of Au-particles is taken equal to 10 nm. The environment medium is air.

In order to interpret the resonant features of the experimental absorption spectra shown in Fig. 2 we applied several theoretical approaches suitable for calculations of the scattering and absorption cross sections of silicon NPs and hybrid silicon-gold NPs. The scattering and absorption spectra of pure spherical Si-particles (Fig. 4a) were obtained in the framework of the Mie theory^13^. Calculations for the Si-particles covered by random clusters of gold nanoparticles (Fig. 4b) were performed within the discrete dipole approximation assuming the presence of random metal-particle clusters (the details of the calculation technique can be found elsewhere)^14^. Dielectric permittivities of Si and Au were taken from^15^ and^16^, respectively. The results of our simulations are shown in Fig. 4. For a pure Si-particle with a diameter of 100 nm (Fig. 4a, the left hand panel) the resonances of the extinction cross section are governed by the excitation of electric dipole (ED) and magnetic dipole (MD) moments of the NP. The resonant absorption at λ ~ 470 nm is associated with MD resonances. This explains the experimental data shown in Fig. 2 for Si (100 nm).

If the size of Si-particles increases, the absorption resonances start to be dominated by high-order multipoles. These are the magnetic quadrupole (MQ) and electric quadrupole (EQ) moments for the pure 200 nm Si-particle (see Fig. 4a, the right-hand panel, λ ~ 490 nm and λ ~ 580 nm). The overlap between these two resonances corresponds to the experimentally observed broad absorption maximum shown in Fig. 2 for Si (200 nm). Covering Si-particles by small Au-particles we affect the spectral distributions of the resonances (Fig. 4b). In this case, the total absorption cross sections increase, and the resonances become broader as compared to the case of pure Si-particles. For particles with 100 nm Si-cores (Fig. 4b, the left-hand panel) one can see the absorption peak at λ ~ 460 nm corresponding to MD excitation and a broad shoulder at λ ~ 530 nm corresponding to the plasmon resonances of Au-NPs. This is in a good agreement with experimental results shown in Fig. 2 for Si-Au (100 nm). The right-hand panel of Fig. 4 shows the calculated spectra of a 200 nm NP. One can see the broad absorption resonance at λ ~ 490–590 nm that corresponds to the overlap between the quadrupole resonances of the Si-core and the plasmon resonance of the Au-NP cluster. The absorption increases at λ ~ 750 nm is due to the combined effect of the MD resonance of Si-core and the plasmon resonances of Au-NP cluster. The calculated spectral dependence of the absorption cross section is very well correlated with experimental observations shown in Fig. 2 for a Si-Au NP of the 100 and 200 nm sizes. Finally, note that the scattering cross sections presented in Fig. 4b have local maxima distributed over the whole considered spectral range. This feature of the scattering spectra is responsible for the different color features of the dark-field images shown in Fig. 2b,c.

## Conclusion

We have applied a method of synthesis of hybrid silicon-gold NPs for producing of 100 and 200 nm hybrid clusters whose optical properties are governed by an interplay of the optical responses of the gold and silicon parts. The developed approach provides an efficient tool of control of the size of hybrid nano-antennas that is paramount for tailoring their optical properties. In colloidal solutions containing Si-Au hybrid particles, strong electric and magnetic multipole resonances have been observed. The possibility of a control of near-field intensity by tailoring the Si-Au hybrid NP size is demonstrated. We have applied several theoretical models which qualitatively reproduce the experimental absorption spectral features. The advantages of hybrid nano-particles for nano-antenna applications are caused by a wide complex of electro-optical characteristics. In particular, the hybrid NPs scatter light of the entire visible range, the intensity of scattering and secondary emission in hybrid clusters is much higher than in individual silicon nanoparticles. Also, the excitation of the Mie-resonance in hybrid clusters can be tailored by tuning the concentrations of gold NPs and the size of a silicon core.
